# Tannic acid attenuates intestinal oxidative damage by improving antioxidant capacity and intestinal barrier in weaned piglets and IPEC-J2 cells

**DOI:** 10.3389/fnut.2022.1012207

**Published:** 2022-11-04

**Authors:** Meiwei Wang, Huijun Huang, Lei Wang, Lanmei Yin, Huansheng Yang, Chiqing Chen, Qiankun Zheng, Shanping He

**Affiliations:** ^1^State Key Laboratory of Developmental Biology of Freshwater Fish, Hunan Provincial Key Laboratory of Animal Intestinal Function and Regulation, Hunan Normal University, Changsha City, China; ^2^Chinese Academy of Science, Institute of Subtropical Agriculture, Research Center for Healthy Breeding of Livestock and Poultry, Hunan Engineering and Research Center of Animal and Poultry Science and Key Laboratory for Agroecological Processes in Subtropical Region, Scientific Observation and Experimental Station of Animal Nutrition and Feed Science in South-Central, Ministry of Agriculture, Changsha City, China; ^3^Wufeng Chicheng Biotechnology Company Limited, Yichang City, China; ^4^Delisi Group Company Limited, Zhucheng City, China

**Keywords:** weaned piglets, IPEC-J2 cells, intestinal antioxidant capacity, intestinal barrier, oxidative stress

## Abstract

Tannic acid (TA) has received widespread attention for its beneficial biological function with antioxidant capacity. This study investigated the protective role of TA on the intestinal antioxidant capacity and intestinal barrier in weaned piglets and porcine intestinal epithelial cells (IPEC-J2). A total of 18 weaned piglets were randomly allocated into two groups (*n* = 9) and fed with a basal diet (control, CON) and a basal diet containing 1,000 mg/kg TA for two weeks. The *in vivo* results showed that treatment with TA increased both glutathione peroxidase (GSH-PX) activity and the protein expression of ZO-1 in the jejunum of weaned piglets, and reduced the level of malondialdehyde (MDA) in the serum and the mRNA and protein expression of Keap1 in the jejunum of weaned piglets. Furthermore, *in vitro* results indicated that TA treatment effectively alleviated tert-butyl hydroperoxide (TBH)-induced oxidative stress in IPEC-J2 cells, improved the antioxidant capacity by elevating the cell redox state and activating the Nrf2 pathway, and improved the intestinal barrier by upregulating the mRNA and protein expression of intestinal tight junction proteins and increasing the transepithelial electrical resistance (TEER) value. In conclusion, these results confirmed that TA relieves oxidative injury and improves intestinal barrier function and intestinal antioxidant capacity by activating the Nrf2 signaling pathway. These findings suggest that TA has the potential application in alleviating oxidative stress in the intestine of weaned piglets.

## Introduction

Early weaning in piglets when they are between 14 days and 30 days is a common practice for elevating commercial profits in pig farming (1). However, early weaning is linked with injury of the gastrointestinal barrier, a decline in metabolism, an increase in disease susceptibility, and then reduced production efficiency (2). It has been reported that numerous factors in early weaning, such as changing environmental cues, can result in oxidative injury by disrupting the balance between the reactive oxygen species (ROS) production and the antioxidant system clearance (3, 4). Oxidative stress damages cellular macromolecules (DNA, lipid, protein) and causes serious oxidative damage and cell death, which leads to adverse effects on animal health and survival (5). Therefore, there is a need to find a beneficial solution to balance the impact of early weaning in pig farming. One such appropriate solution is to supplement natural antioxidants (e.g., plant essential oil, resveratrol, polyunsaturated fatty acid, mineral) in the feed to attenuate the oxidative stress of weaning piglets, and improve their growth performance (6–8).

Tannic acid (TA), as a natural polyphenol, is involved in natural antioxidant activities and is used in various fields (such as agriculture and food). For instance, it is used to maintain meat quality during storage (9). It also has antioxidant and radical scavenging properties which are similar to some antioxidant compounds like Trolox and α-tocopherol and can be used as a food preservative or a nutraceutical (10). As a natural antioxidant, TA could mitigate oxidative injury of the liver by decreasing the content of ROS in Swiss albino mice (11). Some reports found that TA exerts antioxidant activity by forming a complex with iron ions and copper ions (12, 13). In addition, dietary supplementation with TA could improve the antioxidant capacity by increasing the concentration of total antioxidant capacity and glutathione peroxidase (GSH-PX) in the serum and ileum mucosa of weaning piglets, respectively (14). Our previous study confirmed that 2.5 mg/kg TA could attenuate diquat-induced oxidative stress in mice by increasing villus height and activating the Nrf2 signaling pathway (15). In yet another of our studies, we found that dietary supplementation of 1,000 mg/kg TA improved the digestive capacity and gut microbiota of weaned piglets (16). In the current study, we sought to further determine whether TA exerts protective effects against oxidative damage in weaning piglets and IPEC-J2 by enhancing antioxidant capacity and intestinal barrier.

## Materials and methods

### Animals and treatment

The experimental process in the current study was approved according to the Animal Care and Use Committee of Hunan Normal University, Changsha City, Hunan, China (2019 - 1A), and the ethical approval certificate data is shared in the Supplementary materials.

Eighteen weaning piglets [Duroc × (Landrace × Yorkshire), 21 days of age, initial body weight = 5.99 ± 0.13 kg] were randomly assigned to two groups with nine replicate piglets per treatment according to the initial body weight. One group was raised with a corn-soybean meal as the control group (CON) and the other group was fed with 1,000 mg/kg TA based on the corn-soybean meal according to our previous study (16). The dietary composition was designed to follow the nutrient needs of weaned pigs according to the 2012 version NRC standard (Supplementary Table 1). Tannic Acid was obtained from the Wufeng Chicheng Biotechnology Company Limited, Yichang, China (extracted from Chinese gallnut). It was microencapsulated by Kangjude Company Limited, Hangzhou, China, and its effective concentration was 30% after microencapsulation. The piglets were housed in pens (0.5 × 1 m) equipped with feeders and water (25°C ± 2°C with an average humidity of 65 ± 5%) and could freely eat the provided diet and drink water for 14 days.

### Sample collection

On day 14 of the experiment, all the piglets were fasted for 12 h, then using the method of complete anesthesia, the piglets were injected with 4% sodium pentobarbital solution (40 mg/kg BW) for euthanasia. We collected blood and tissue samples according to our previous study (16). Jejunal mucosa and ileal mucosa were scraped by microslides and quickly frozen in liquid nitrogen, and blood samples were centrifuged with 845 g for 10 min to get the serum and stored at −80°C. Serum and mucosa of the jejunum and ileum were used for measuring the enzyme activity of antioxidant capacity and the level of malondialdehyde (MDA). Moreover, the mucosa of the jejunum and ileum were also used for detecting gene expression and protein expression.

### Measurement of MDA contents and antioxidative enzyme activity

The samples of mucosa and serum were detected as previously described (15). Briefly, the content of MDA and enzymatic activities of superoxide dismutase (SOD), catalase (CAT), glutathione peroxidase (GSH-PX), total antioxidant capacity (T-AOC), and total glutathione were measured according to the manufacturers' instructions of commercially available products (Nanjing Jiancheng Bioengineering Institute, Nanjing, China).

### Gene expression analysis

The gene expression analysis in this study was performed as previously described (16). Briefly, RNA was extracted using Trizol reagent (Takara, Tokyo, Japan) and 1.0 μg of RNA was reverse transcribed to cDNA using the cDNA synthesis kit (Takara, Tokyo, Japan). Primers in this study were designed on the website of the National Center for Biotechnology Information (Supplementary Table 2). Real-time PCR (RT-PCR) was performed as previously described (17). SYBR Green mix (Thermo Scientific, Waltham, USA) was used in the RT-PCR analysis. Each sample was measured for three reactions. The gene of β-actin was used as an internal control to standardize the target gene expression. The relative mRNA expression was quantified with the comparative Ct value calculation.

### Western blotting analysis

Western blotting analysis in this study was performed as previously described (15). Antibodies against Claudin-1, Occludin, β-actin, glutathione peroxidase4 (Gpx4), and horseradish peroxidase-linked secondary antibodies were bought from bimake (Shanghai, China). Antibodies against nuclear factor erythroid 2-related factor 2 (NRF2), zonula occludens-1 (ZO-1), and Kelch like-ECH-associated protein 1 (KEAP1) were bought from Proteintech (Wuhan, China). Antibodies against LC3 and Sequestosome 1 (p62/SQSTM1) were bought from Abclonal (Wuhan, China).

### Cell culture

The intestinal porcine epithelial cell of the jejunum (IPEC-J2, obtained from Institute of Subtropical Agriculture, Chinese Academy of Sciences) was cultured in high-glucose (25 mM) Dulbecco's modified Eagle's medium (DMEM, Gibco, USA) and supplemented with 10% fetal bovine serum (Biological Industries, Israel) and 1% antibiotics of penicillin-streptomycin (Gibco, USA). Cells were cultured in a humidified incubator (37°C, 5% CO_2_).

### Cell viability analysis

Cell viability was measured by cell-counting kits CCK-8 (Dojindo, Osaka, Japan). The TA used for the cell treatment assay was bought from Sigma (Sigma, Mo, USA). Cells were plated in 96-well plates (10,000/well) and kept in DMEM for 24 h. The cells were treated with different concentrations of TA for 12 h or treated with different concentrations of tert-butyl hydroperoxide (TBH; Sigma, Mo, USA) for 3 h. Moreover, to analyze the beneficial effect of TA on cell viability, cells were pretreated with different concentrations of TA for 12 h, and then cells were treated with TBH at the concentration of 200 μM for 3 h except for the control group. After the treatment, 10 μl CCK-8 reagent was added to the cells of each well for 2 h, and then the optical density of 450 nm was detected by an enzyme-linked detector (BioTek Instruments, Winooski, USA).

### Scratch wound assay

Cells were plated in a six-well-plate and cultured in the incubator. When the cell growth filled the compact monolayer, a scratch was acquired with a cleaned pipette tip (200 μl). The monolayer cells were gently washed with PBS for three times to remove excess detached cells. Monolayer cells were treated with different concentrations of TA. After 0, 12, 18, and 24 h of treatment, images were pictured using inverted light microscopy (Leica, Germany). Quantification of the width with the wound closure was measured using the image pro plus software to determine the width devoid of cells at 0 h and determined at 12, 18, and 24 h. The width was reduced from the 0 h area and divided by the 0 h width, to get a percentage to show the degree of wound closure.

### Measurement of transepithelial electrical resistance

Cells were plated in cell culture transwells (Corning, NY, USA) with a six-well plate, which has a pore size of 0.4 μM and a membrane area of 1.12 cm^2^. When the cells formed the monolayer integrity, they were measured with the stable value of transepithelial electrical resistance (TEER), and were treated with TA and TBH. The cells were treated with different concentrations of TA, and the TEER value was measured using Millicell Electrical Resistance System (Millipore, Molsheim, USA) at different time points of treatment. Moreover, cells were pretreated with different concentrations of TA for 12 h followed by treatment with 200 μM TBH for 3 h except for the control group and used to determine the TEER value.

### Determination of ROS content

The content of ROS in cells was measured with the ROS measurement commercial product (Beyotime, China) according to the manufacturer's instructions. Initially, cells were gathered in tubes at the end of treatments and incubated with DCFH-DA dye (10 μM in serum-free DMEM) and placed in dark condition for 20 min at 37°C. Subsequently, the DCFH-DA dye was washed, and cells were re-suspended in PBS after washing three times by FBS-free DMEM. Finally, the concentration of ROS in cells was measured by flow cytometry (Thermo Scientific, Waltham, USA).

### Statistical analysis

Data from animal experiments were analyzed by *t*-test. Data from cell treatment were analyzed by one-way ANOVA and the differences among different groups in cell treatment were analyzed with Duncan's range test. All data were analyzed using version 22 of the SPSS software (SPSS Inc., Chicago, USA) and indicated as mean ± SEM. The results were considered statistically significant at *P*-values < 0.05, while 0.05 ≤ *P*-values < 0.10 were considered as the trend for statistically significant.

## Results

### Effect of TA on the activities of antioxidant enzymes and the content of MDA in weaned piglets

As shown in Tables 1, 2, the jejunal enzyme activity of GSH-PX was significantly elevated, and the content of MDA and ileal enzyme activity of SOD tended to decrease in the TA group of pigs. In addition, the content of MDA in serum was lower in the TA treatment than in the CON group. No differences were found in the activities of other antioxidant enzymes between the two groups.

**Table 1:** The jejunal and ileal antioxidant capacity of weaning piglets^1^.

**Item**	**Dietary treatment**		***P*-value**
	**CON**	**TA**	
**CAT**, **μg/mg of protein**			
Jejunum	5.88 ± 0.70	4.78 ± 0.75	0.803
Ileum	3.49 ± 0.58	3.79 ± 0.42	0.471
**GSH-PX**, **μmol/μg of protein**			
Jejunum	16.43 ± 2.72^b^	27.27 ± 2.77^a^	0.024
Ileum	35.37 ± 3.45	40.20 ± 3.15	0.322
**MDA, nmol/mg of protein**			
Jejunum	1.47 ± 0.17	1.41 ± 0.71	0.867
Ileum	1.26 ± 0.05	1.10 ± 0.05	0.058
**SOD**, **μg/mg of protein**			
Jejunum	53.95 ± 3.75	51.35 ± 4.44	0.511
Ileum	50.71 ± 3.74	40.30 ± 3.42	0.063

1 Values are expressed as mean ± SEM; n = 9.

CON, basic diet; TA, a basic diet supplemented with 1,000 mg/kg tannic acid.

a, b values in the same row not sharing a common superscript differ significantly.

**Table 2:** The antioxidant capacity of serum in weaning piglets^1^.

**Item**	**Dietary treatment**		***P*-value**
	**CON**	**TA**	
CAT, U/ml	46.03 ± 11.86	30.55 ± 6.78	0.606
GSH-PX, U/ml	443.06 ± 4.76	437.67 ± 2.16	0.506
SOD, U/ml	19.14 ± 0.63	19.87 ± 0.80	0.397
MDA, nmol/ml	4.76 ± 0.51^a^	3.17 ± 0.18^b^	0.035

1 Values are expressed as mean ± SEM; n = 9.

CON, basic diet; TA, a basic diet supplemented with 1,000 mg/kg tannic acid.

a, b values in the same row not sharing a common superscript differ significantly.

### Effect of TA on the Nrf2-keap1 pathway in weaned piglets

**Figure 1:**
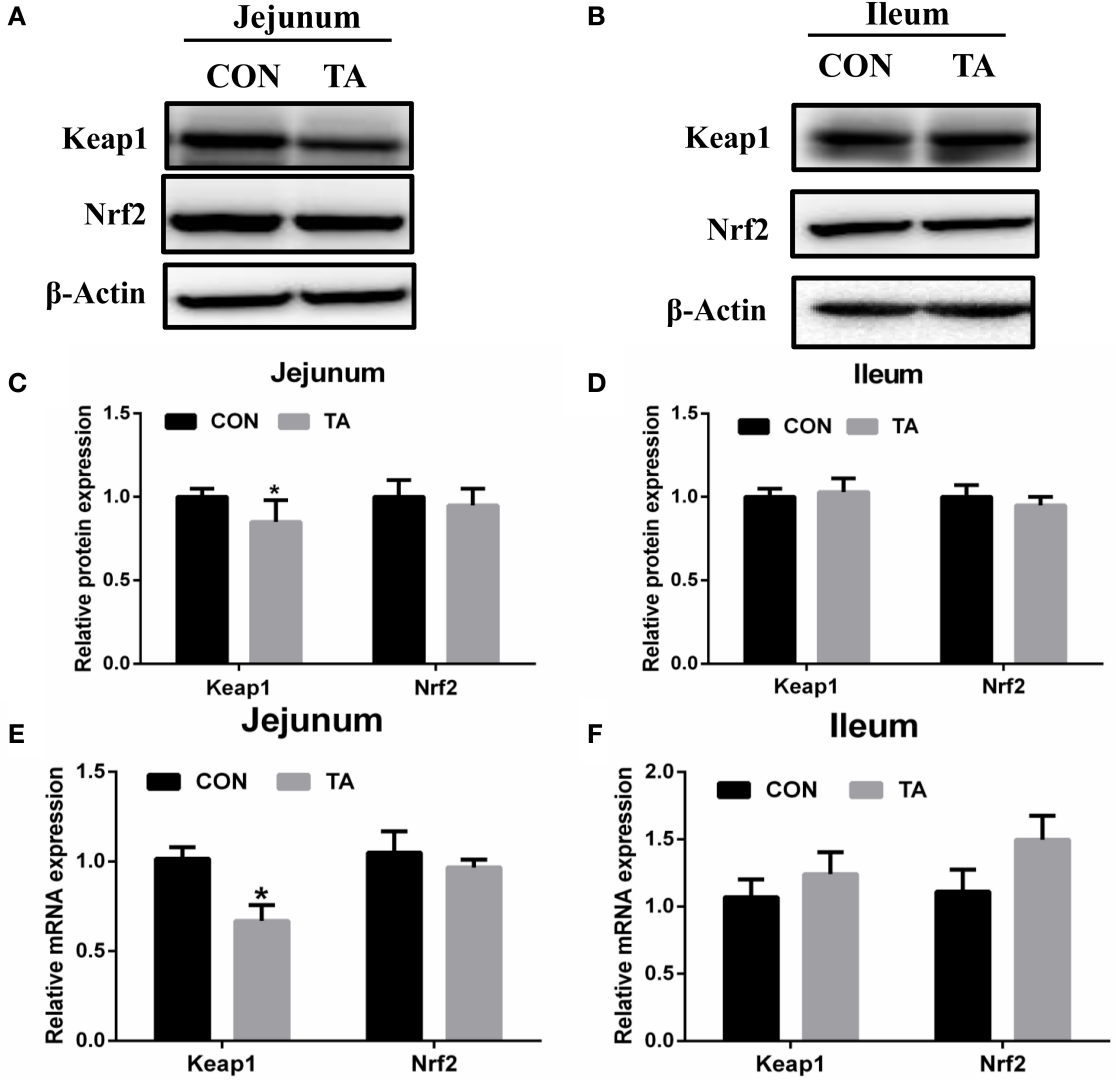
Effects of tannic acid (TA) on the Nrf2-Keap1 pathway in weaning piglets. The relative protein levels of the Nrf2-Keap1 pathway in the jejunum **(A)** and in the ileum **(B)**. Quantitative analysis of the relative protein levels of Keap1 and Nrf2 in the jejunum **(C)** and in the ileum **(D)**. The relative mRNA levels of Keap1 and Nrf2 in the jejunum **(E)** and in the ileum **(F)**. CON, basic diet; TA, a basic diet supplemented with 1,000 mg/kg tannic acid. Data are presented as mean ± SEM, *n* = 9. *P < 0.05. * means comparing to the CON group.

The mRNA and protein expression of Keap1 was significantly reduced in the jejunum of the TA group of weaned piglets compared with weaned piglets fed with the CON diet (Figure 1). However, the expression of Nrf2 remained unaltered in the jejunum and ileum between the two groups.

### Effect of TA on the intestinal tight junction of weaned piglets

**Figure 2:**
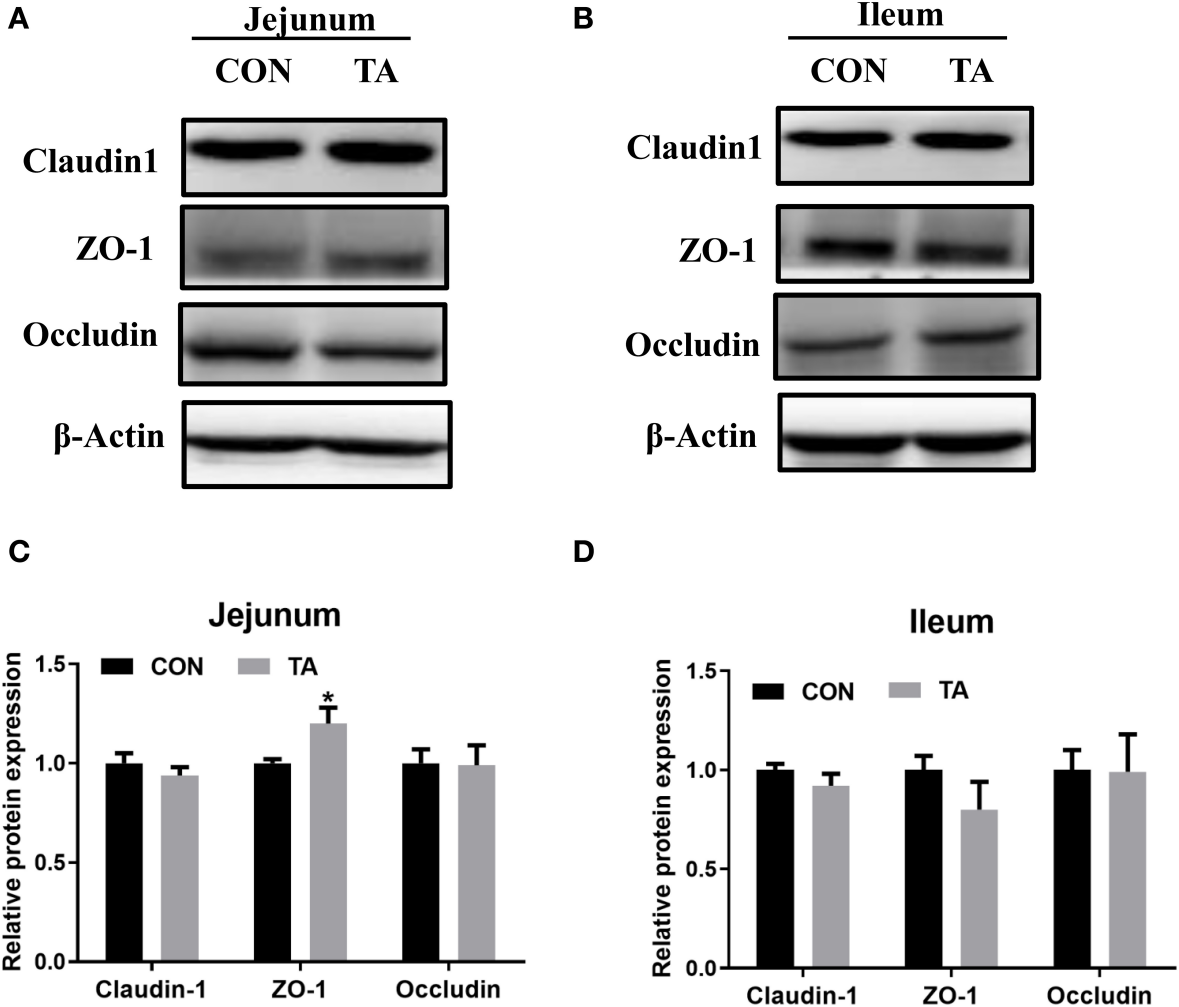
Effects of tannic acid (TA) on the intestinal tight junction in weaning piglets. The relative protein levels of intestinal tight junction proteins in the jejunum **(A)** and in the ileum **(B)**. Quantitative analysis of the relative protein levels of intestinal tight junction proteins in the jejunum **(C)** and in the ileum **(D)**. CON, basic diet; TA, a basic diet supplemented with 1,000 mg/kg tannic acid. Data are presented as mean ± SEM, *n* = 9. *P < 0.05. * means comparing to the CON group.

The protein expression of ZO-1 was increased in the jejunum of the TA group compared with the CON group (Figure 2). The levels of other intestinal tight junction proteins were not altered between the two groups.

### Effect of TA on cell viability of TBH-treated IPEC-J2 cells

**Figure 3:**
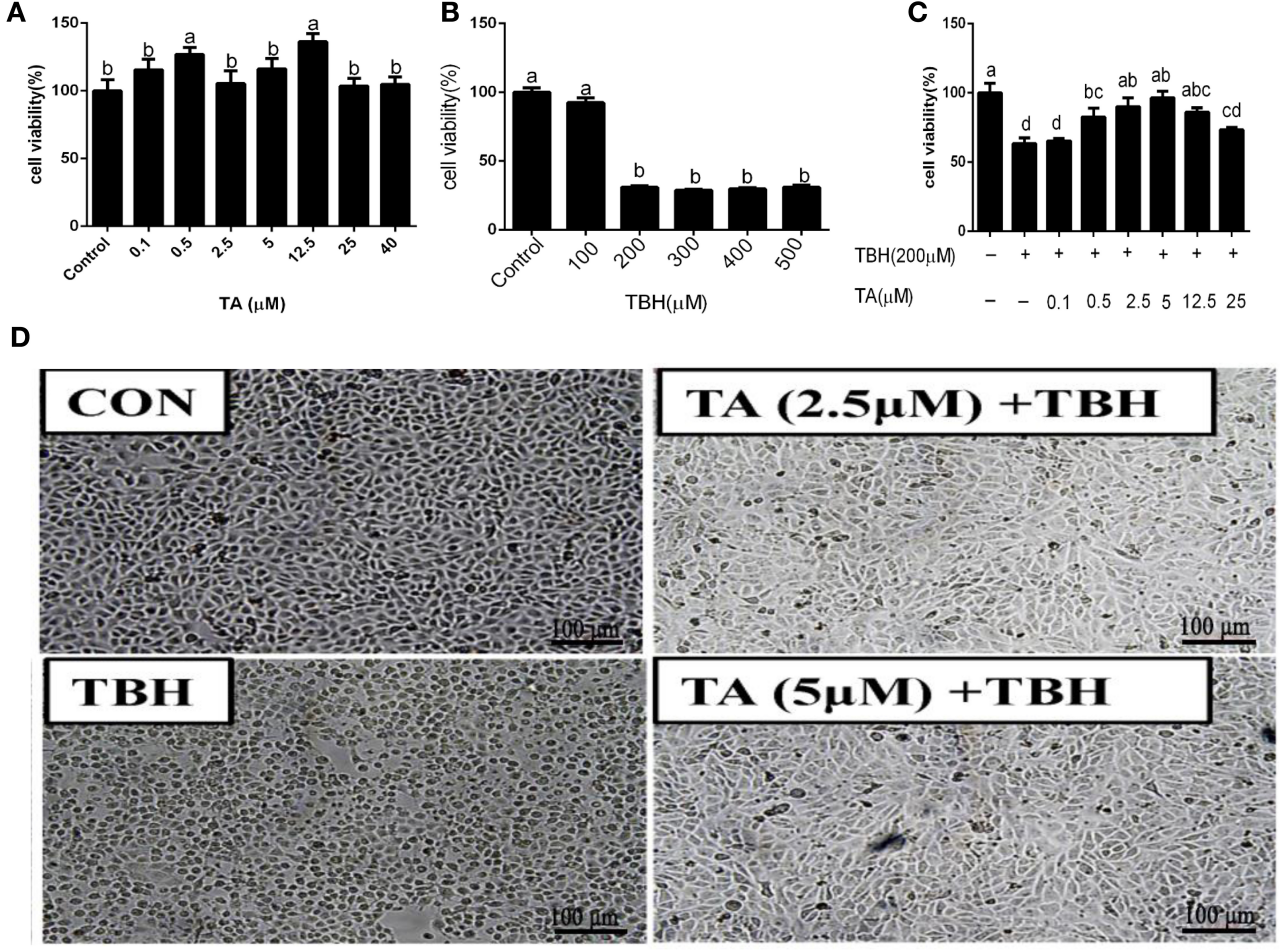
Effects of tannic acid (TA) and tert-butyl hydroperoxide (TBH) on the cell viability of IPEC-J2 cells. Cells were treated with different doses of TA for 12 h **(A)**, treated with different doses of TBH for 3 h **(B)**, or pre-treated with different doses of TA for 12 h and treated with or without 200 μM TBH for 3 h **(C)**, and then assayed for cell viability. The representative picture of the morphology of IPEC-J2 cells treated with TA and TBH at 100 × magnification **(D)**. Data are presented as mean ± SEM, *n* = 6 for each group. TA, tannic acid; TBH, tert-butyl hydroperoxide. ^a, b^values in the same row not sharing a common superscript differ significantly.

The viability of cells treated with 0.5 and 12.5 μM TA was significantly increased compared with the control group and other TA-treated concentration groups (Figure 3A). To delineate the mechanism by which TA attenuates oxidative damage in the intestine, we established the oxidative stress model of intestinal epithelial cells induced by TBH. The results showed that the viability of cells treated with 200 to 500 μM TBH for 3 h was significantly decreased compared with the control group (Figure 3B). Hence, we treated IPEC-J2 cells with 200 μM TBH for 3 h for the following experiments to induce oxidative damage of IPEC-J2 cells. As seen in Figure 3C, treatment with concentrations of 0.5–12.5 μM TA could relieve the decrease of cell viability caused by TBH. Also, as seen in Figure 3D, TA treatment could alleviate the change in cell morphology caused by TBH. Thus, the appropriate TA concentrations for the following experiments were chosen to be 2.5 and 5 μM.

### Effect of TA on the redox balance and Nrf2 pathway of IPEC-J2 cells

**Figure 4:**
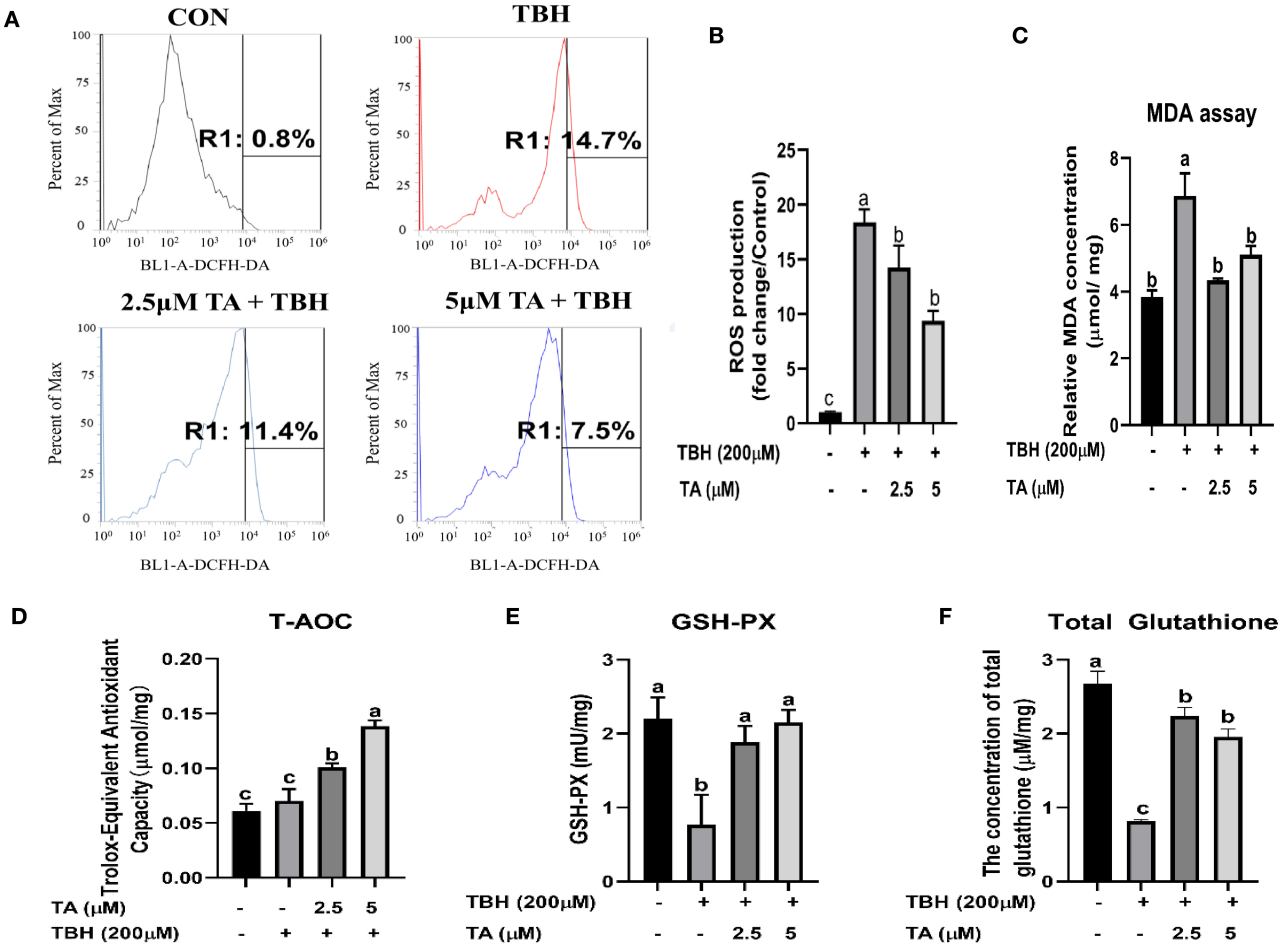
Effects of tannic acid (TA) on the redox state of IPEC-J2 cells. IPEC-J2 cells were treated with indicated doses of TA for 12 h followed by TBH (200 μM) for 3 h. Flow cytometry analysis data of the cellular ROS levels are shown in a gate information **(A)**. Quantification of the cellular ROS levels from flow cytometry data **(B)**. The concentration of the cellular MDA **(C)** and total glutathione **(F)** and the antioxidant enzyme activities of T-AOC **(D)** and GSH-PX **(E)** were determined. Data are presented as mean ± SEM, *n* = 3. TA, tannic acid; TBH, tert-butyl hydroperoxide. ^a, b^values in the same row not sharing a common superscript differ significantly.

**Figure 5:**
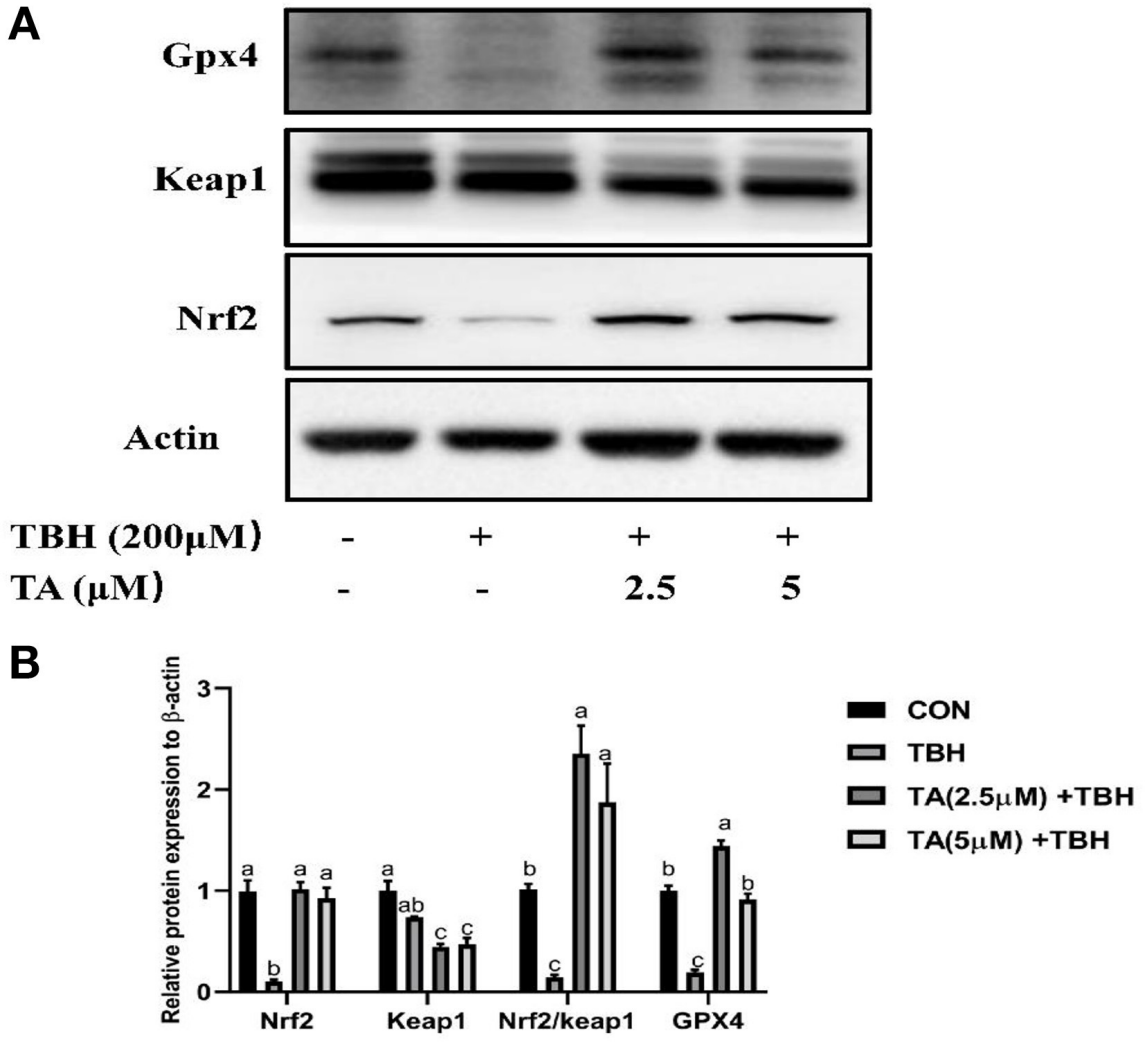
Effects of tannic acid (TA) on the Nrf2 signaling pathway of IPEC-J2 cells. Western blotting analysis of the protein expression of Nrf2, Keap1, and GPX4 is shown **(A)**. Quantitative analysis of the protein expression of Nrf2, Keap1, and GPX4 **(B)**. Data are presented as mean ± SEM, *n* = 3. TA, tannic acid; TBH, tert-butyl hydroperoxide. ^a, b^values in the same row not sharing a common superscript differ significantly.

To explore whether TA treatment regulates the redox balance of cells, we analyzed the level of MDA and ROS, detected the activity of antioxidative enzymes, and measured the protein abundance of the Nrf2 pathway in cells. As shown in Figures 4A,B, TA treatment (2.5 and 5 μM) relieved TBH-induced increase of ROS content in cells. Furthermore, TA treatment (2.5 and 5 μM) also decreased the content of MDA, which was induced by TBH treatment in IPEC-J2 cells (Figure 4C). Moreover, TA treatment (2.5 and 5 μM) could effectively elevate the content of T-AOC, GSH, and total glutathione, which were downregulated by TBH treatment in IPEC-J2 cells (Figures 4D–F). Furthermore, we explored whether TA attenuates oxidative damage by the regulation of the Nrf2 pathway. The results showed that TA treatment (2.5 and 5 μM) could increase the protein abundance of Nrf2 and GPX4 and decrease the protein abundance of Keap1 in TBH-treated cells (Figure 5).

### Effect of TA on the wound healing and TEER value of the IPEC-J2 cells

**Figure 6:**
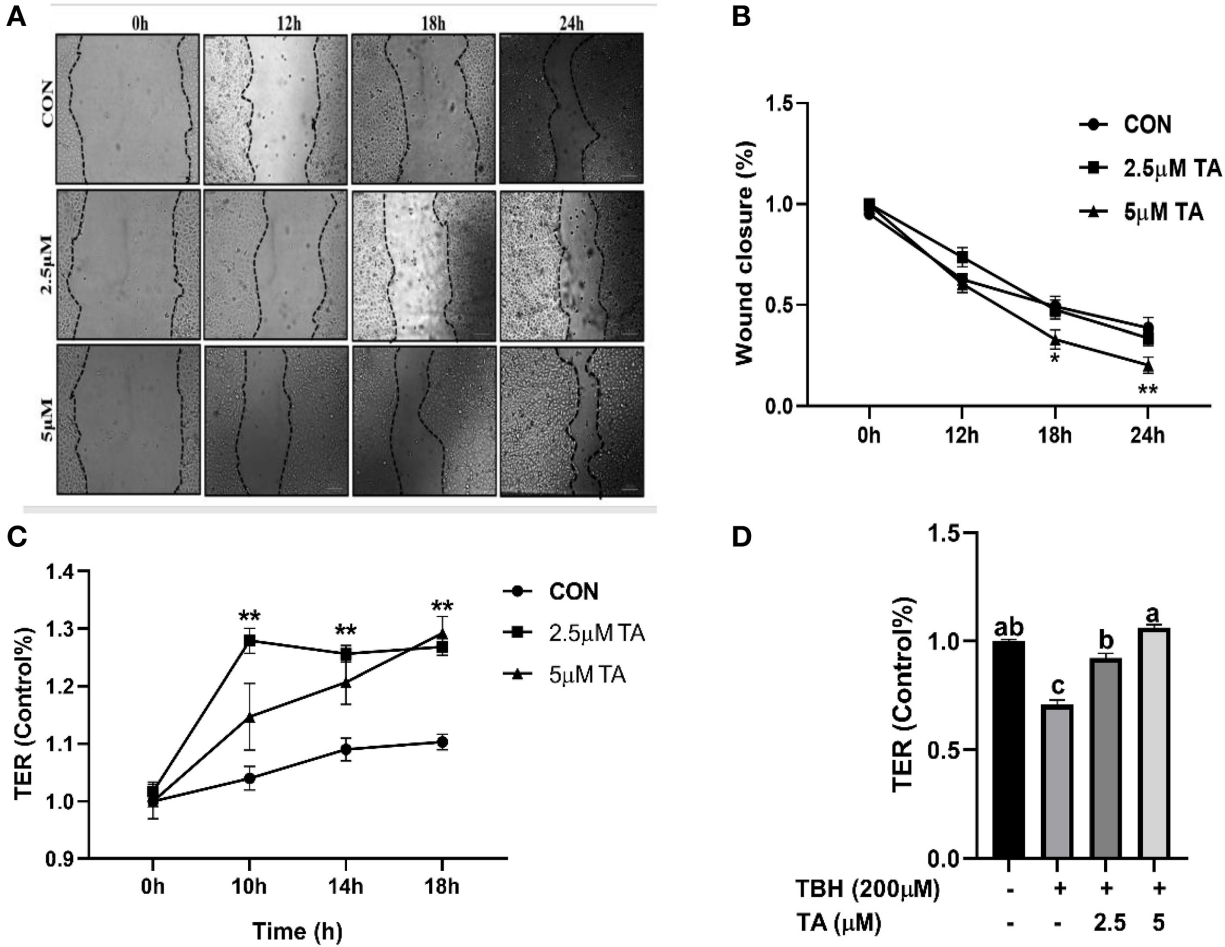
Effects of tannic acid (TA) on wound healing and transepithelial electrical resistance (TER) of IPEC-J2 cells. The representative images of wound healing at 100 × magnification **(A)**. Quantitative analysis of the width of wound **(B)**. Effect of TA on the TER value in IPEC-J2 cells **(C)**. Effect of TA on the TER value in TBH-treated IPEC-J2 cells **(D)**. Data are presented as mean ± SEM, *n* = 3. ^a, b^values in the same row not sharing a common superscript differ significantly. TA, tannic acid; TBH, tert-butyl hydroperoxide. * *P* < 0.05, 0.01 < **P < 0.05.

As shown in Figures 6A,B, the TA group (5 μM) had a smaller width wound than the control group and the TA group (2.5 μM) at both 18 h and 24 h. The TA treatment (2.5 and 5 μM) upregulated the TEER values of cells and alleviated the TBH-induced decrease in the TEER value of cells (Figures 6C,D).

### Effect of TA on the intestinal tight junction of IPEC-J2 cells

**Figure 7:**
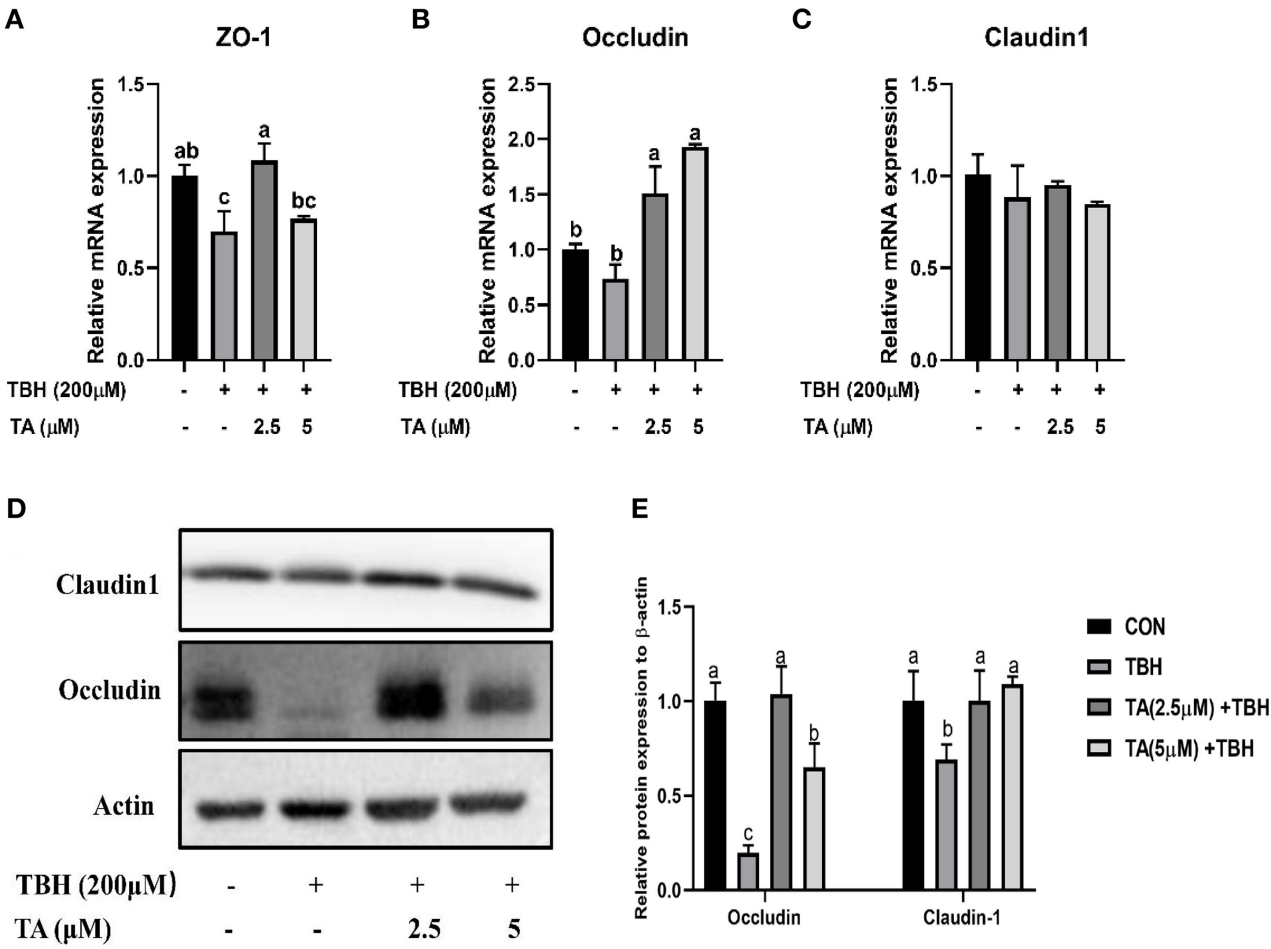
Effects of tannic acid (TA) on the tight junction of IPEC-J2 cells. The mRNA expression of ZO-1 **(A)**, Occludin **(B)**, and Claudin-1 **(C)**. Western blotting analysis of the protein expression of Occludin and Claudin-1 is shown **(D)**. Quantitative analysis of the protein expression of Occludin and Claudin-1 **(E)**. Data are presented as mean ± SEM, *n* = 3. TA, tannic acid; TBH, tert-butyl hydroperoxide. ^a, b^values in the same row not sharing a common superscript differ significantly.

To explore whether TA treatment attenuates the intestinal barrier injury of TBH-treated cells, we analyzed the relative mRNA and protein abundance of intestinal tight junction proteins in IPEC-J2 cells. The results showed that the mRNA expression of Occludin and increased protein abundance of Claudin-1 and Occludin were remarkably increased in the pretreated TA groups (2.5 and 5 μM) compared with the TBH group. Moreover, the mRNA abundance of ZO-1 and the protein abundance of Occludin were elevated in the pretreated TA groups (2.5 μM), compared with the TBH group and the treated TA groups (5 μM; Figure 7).

### Effect of TA on the mRNA expression of inflammatory factors and autophagy in IPEC-J2 cells

**Figure 8:**
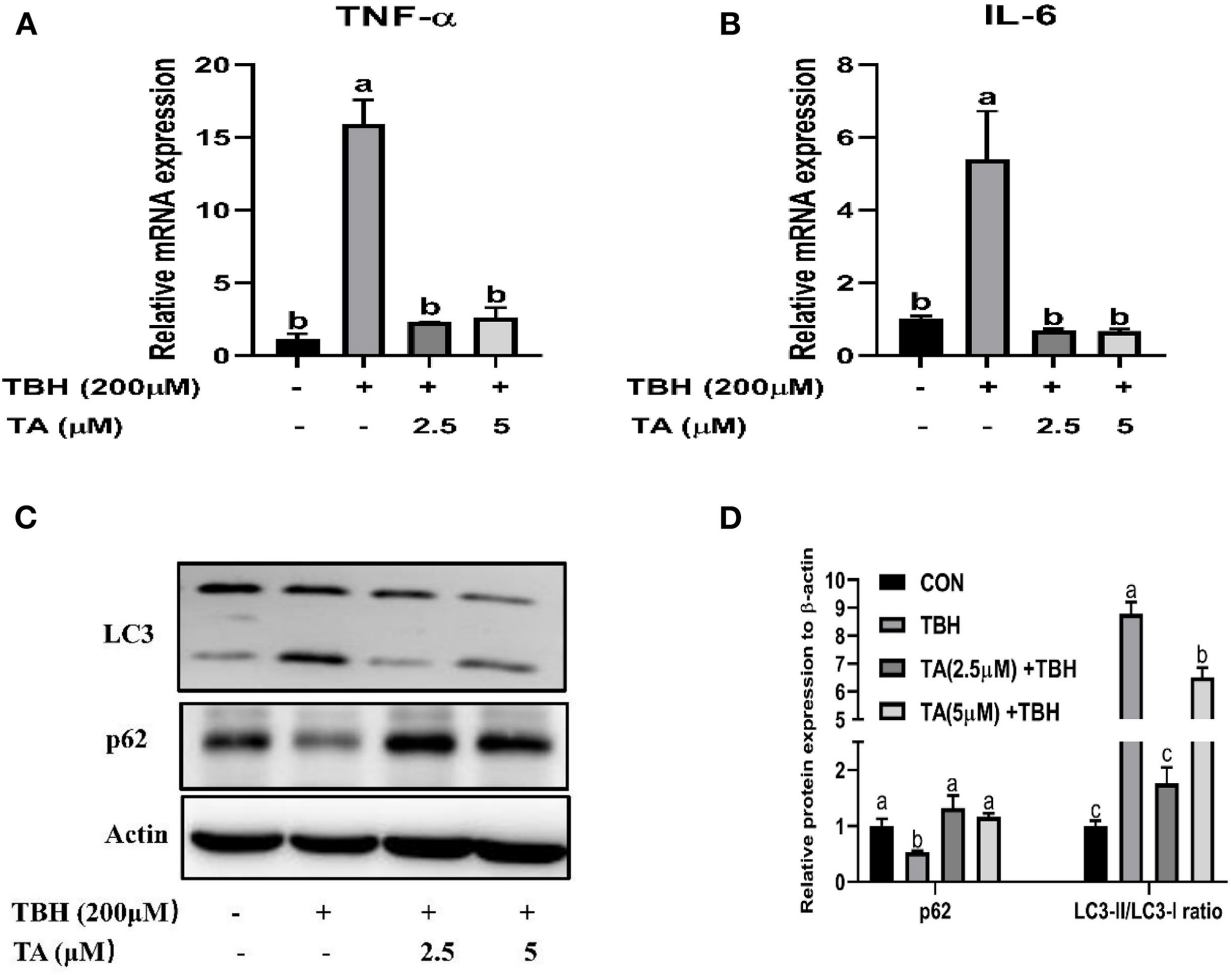
Effects of tannic acid (TA) on the inflammatory cytokines and autophagy of IPEC-J2 cells. The mRNA expression of TNF-α **(A)** and IL-6 **(B)**. Western blotting analysis of the protein expression of LC3 and p62 is shown **(C)**. Quantitative analysis of the protein expression of LC3 and p62 **(D)**. Data are presented as mean ± SEM, *n* = 3. TA, tannic acid; TBH, tert-butyl hydroperoxide. ^a, b^values in the same row not sharing a common superscript differ significantly.

To further determine whether TA ameliorates TBH-induced inflammation and autophagy, we examined the mRNA abundance of inflammatory factors and the protein abundance of autophagy-related proteins. The results indicated that TA treatment suppressed the mRNA abundance of IL6 and TNF-α induced by TBH treatment in IPEC-J2 cells (Figures 8A,B). Furthermore, TA treatment also altered the protein abundance of LC3 and SQSTM/P62 in TBH-treated IPEC-J2 cells (Figures 8C,D).

**Figure 9:**
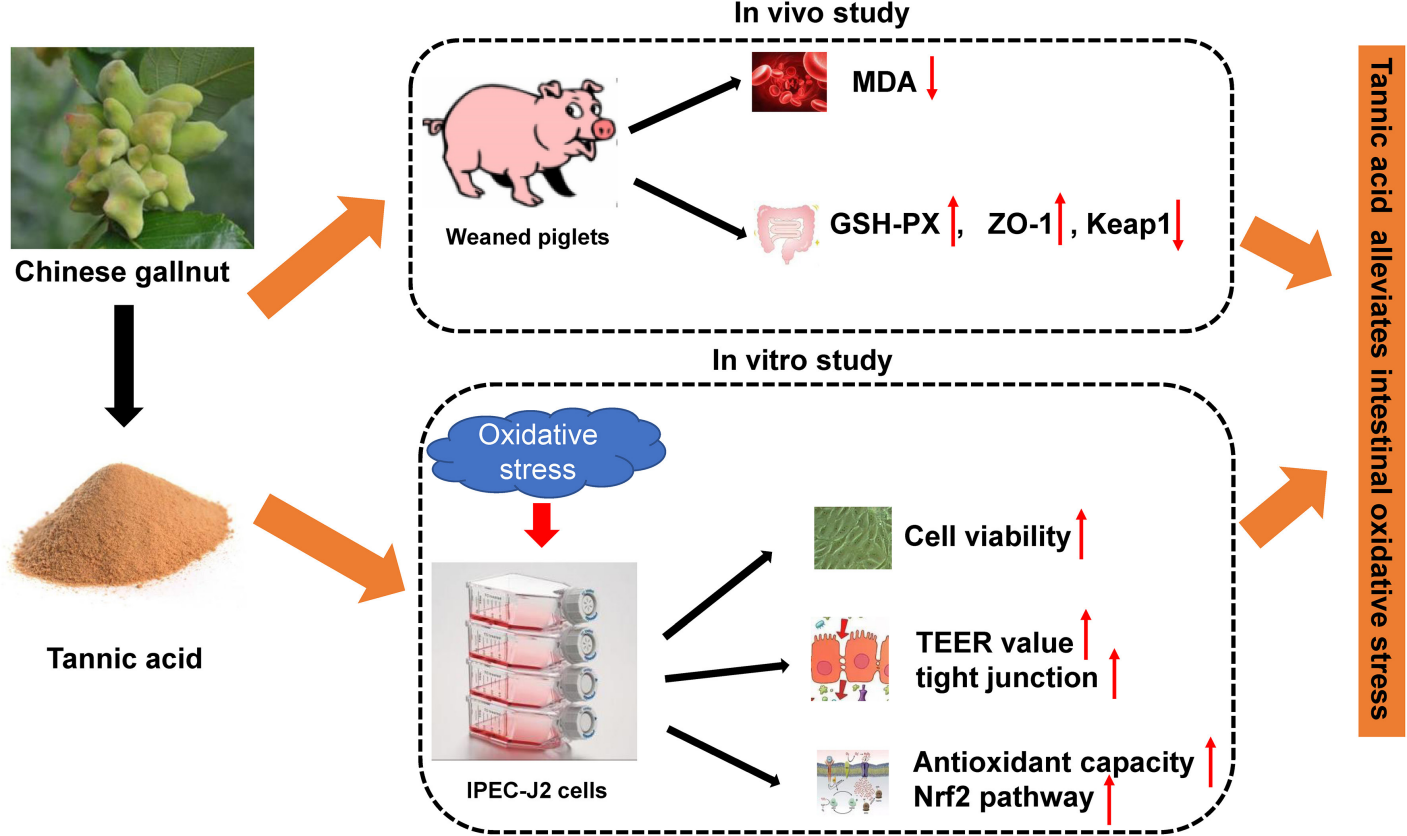
A model of tannic acid alleviating intestinal oxidative stress.

## Discussion

Weaning stress disrupts the intestinal redox balance and damages the intestinal barrier in pigs, such as the increase of MDA concentration in plasma, the inhibition of antioxidative enzyme activity in plasma, the decrease of antioxidative gene expression in the jejunum, and the suppression of the Nrf2 signaling pathway in the jejunum (4). It is common for some polyphenolic secondary metabolites from plants to display their antioxidant properties (18). Tannic acid, as a kind of natural polyphenolic secondary metabolite, has long been considered an antioxidant and has been widely used in the food industry and medical field (19, 20). Similar to the livestock field, some research has shown that TA displays a powerful antioxidative property. A recent study has reported that dietary supplementation of 1,000 mg/kg hydrolyzable tannins improves the serum and intestinal mucosa antioxidant capacity in weaning piglets (14). Moreover, another study has shown that TA effectively suppresses paraquat-induced lipid peroxidation of the liver and lung in mice (21). Consistent with these findings, the current results showed that dietary supplementation of TA could decrease the content of MDA in serum and increase the activity of GSH-PX in the jejunum of weaned piglets.

To further elucidate the protective role of TA on intestinal injury in cells, we used TBH as a prooxidant reagent to incur oxidative damage in cells as previously described (22). In the current study, TA treatment could relieve the decrease of cell viability challenged by TBH and maintain normal cell morphology. Furthermore, *in vitro* study also confirmed that the TA treatment decreased the content of ROS and MDA and enhanced the enzyme activities of T-AOC, GSH-PX, and total glutathione in TBH-treated cells. Consistent with *in vivo* data, our data displayed that TA could elevate the antioxidant capacity by increasing the activities of antioxidant enzymes and decreasing the oxidative products in weaned piglets and TBH-treated IPEC-J2.

The Nrf2 signaling pathway is involved in the body to defend against oxidative damage, which regulates the expression of a list of antioxidant genes to enhance cellular antioxidant capacity (23, 24). In the situation of oxidative stress in the cells, the Nrf2-Keap1 complex is dissociated, Keap1 is degraded by the proteasome, and Nrf2 translocates to the cellular nucleus, and enhances the cell antioxidant capacity (25). Our data revealed that TA treatment could decrease the jejunal mRNA abundance and protein abundance of Keap1 in weaned piglets, while it did not effectively upregulate the expression of Nrf2. It was reported that TA ameliorates arsenic trioxide-induced oxidative damage, apoptosis, and inflammation by modulating the NF-kappa B/Nrf2 pathway in rats (26). In addition, the TA treatment significantly increased the protein abundance of Nrf2 and heme oxygenase-1 against traumatic brain injury in a rodent model (27). To further investigate the underlying mechanism by which TA modulates the Nrf2 pathway, we used the TBH-challenged IPEC-J2 cell model to perform the experiments. *In vitro* study confirmed that TA could stimulate the Nrf2 signaling pathway and upregulate the downstream protein abundance of GPX4 in IPEC-J2 cells, which could effectively alleviate TBH-induced oxidative stress. Collectively, these results support the view that TA can enhance the antioxidant capacity by activating the Nrf2 pathway (28, 29).

Dysfunction of the intestinal barrier can be caused by several types of stresses, such as environmental change, nutritional change, pathological condition, or a combination of these (30). A previous study has indicated that weaning stress induces the expression of tight junction proteins, and increases intestinal permeability in weaned piglets (31). In the present study, our results showed that TA treatment could elevate the jejunal protein expression of tight junction (ZO-1) in weaned piglets, which was consistent with other studies (32). Yet, more work is needed to perform the electrophysiological and permeability analysis of the intestinal tissues to further confirm the beneficial effect of TA on the intestinal barrier.

The intact intestinal barrier can protect gut health, but stresses destroy the integrity of the intestinal barrier. Wound healing of epithelial cells is self-repaired through the proliferation and migration of epithelial cells, thus the self-repair ability of intestinal epithelial cells plays a vital role in maintaining the integrity of the intestinal barrier (33, 34). Natural products (such as essential oils, tannins, and saponins) have anti-inflammatory, antioxidant, and antibacterial properties, and thus can be used as would healing agents (35). Using the scratch wound assay of IPEC-J2 cells, our data indicated that treated with 5 μM TA could accelerate the self-repair of wound healing in cells. Furthermore, our data showed that TA treatment increased the TEER value and prevented the decline of TEER value in IPEC-J2 cells challenged with TBH. Similarly, Bianchi et al. (36) reported that the active component of tannis (catechin and procyanidin B-2) has a positive effect on intestinal barrier function as indicated by increased TEER value in human intestinal cell monolayers.

Apart from the TEER value and wound healing, the expression of tight junction proteins is also crucial for maintaining an integrated intestinal epithelial layer. Therefore, we determined the mRNA expression and protein expression of intestinal tight junction proteins (ZO-1, Claudin1, and Occludin) in IPEC-J2 cells and found that TA treatment prevented TBH-induced decrease of protein abundance of Claudin-1 and Occludin and the mRNA expression of ZO-1 and Occludin, which is consistent with our previous study (15). These results indicated that TA can effectively improve the intestinal barrier in weaned piglets and intestinal epithelial cells. The localization of tight junction proteins needs to be determined to support the protective role of TA in the intestinal barrier of epithelial cells.

To further elucidate the effect of TA on intestinal oxidative stress status, we examined inflammation and autophagy *in vitro*. It has been displayed that oxidative damage leads to inflammation by activating a variety of transcription factors of inflammatory pathways and inducing the expression of various inflammatory gene products (such as pro-inflammatory cytokines IL-1, IL-6, and the chemokine IL-8). Tannic acid, which contains numerous biological activities of polyphenols, can modulate the inflammatory processes (37, 38). Our results indicated that the expression of the inflammatory cytokines IL-6 and TNF-α in IPEC-J2 cells was induced by TBH, while TA treatment could alleviate the increase of inflammatory cytokines, suggesting that TA can attenuate the inflammation induced by oxidative stress. Furthermore, under the conditions of oxidative stress, an aberrant increase of reactive oxygen species in the tissue can incur autophagy *via* multiple signal pathways (39). An increase in LC3-II expression is considered to be a marker of autophagy. Dysregulation of autophagy was indicated to induce the activation of the Nrf2 signaling pathway in a p62-dependent manner, which allows p62 to sequester Keap1 into the autophagosomes (40). In this study, our data also indicated that TA treatment could decrease the ratio of LC3- II to LC3- I, and the protein abundance of p62 correlated positively with the Nrf2 pathway in cells challenged with TBH. It was shown that TA could inhibit autophagy and stimulate the Nrf2 signal pathway to alleviate oxidative damage in IPEC-J2 cells.

In conclusion, supplementation of 1,000 mg/kg TA can elevate the protein abundance of ZO-1 and enhance the antioxidant capacity by enhancing the activity of GSH-PX in the jejunum and reducing the content of MDA in the serum of weaning piglets. Our *in vitro* results also showed that TA treatment can improve cell viability, cell antioxidant enzyme, TEER value, and intestinal tight junction and stimulate the Nrf2 pathway against oxidative injury in IPEC-J2 cells. Taken together, the current study indicated that TA, as a plant extract, attenuates oxidative stress by enhancing the antioxidant capacity and intestinal barrier in weaning piglets and IPEC-J2 cells.

## Data availability statement

The original contributions presented in the study are included in the article/Supplementary material, further inquiries can be directed to the corresponding author.

## Ethics statement

The animal study was reviewed and approved by the experimental design and programming were supervised and permitted by the Animal Care and Use Committee of Hunan Normal University, Changsha City, Hunan, China (2019-1A).

## Author contributions

MW: methodology, investigation, data curation, writing—original draft, and funding acquisition. HH and LW: investigation and data curation. LY: investigation and writing—review and editing. HY: validation and data curation. CC and QZ: investigation and resources. SH: methodology, supervision, writing—review and editing, and funding acquisition. All authors contributed to the article and approved the submitted version.

## Funding

This study was supported by the Hunan Science and Technology Project (2017XK2020), the Special Funds for Construction of Innovative Provinces in Hunan Province (2019RS3022), the Key R&D Program of Hunan Province (2019NK2161), the Hunan Provincial Innovation Foundation for Postgraduate (CX20200527), and the Xiaoxiang Scholar Distinguished Professor Fund of Hunan Normal University.

## Conflict of interest

Author CC was employed by Wufeng Chicheng Biotechnology Company Limited. Author QZ was employed by Delisi Group Company Limited. The remaining authors declare that the research was conducted in the absence of any commercial or financial relationships that could be construed as a potential conflict of interest.

## Publisher's note

All claims expressed in this article are solely those of the authors and do not necessarily represent those of their affiliated organizations, or those of the publisher, the editors and the reviewers. Any product that may be evaluated in this article, or claim that may be made by its manufacturer, is not guaranteed or endorsed by the publisher.
