# Supplementary material for: Tannic acid attenuates intestinal oxidative damage by improving antioxidant capacity and intestinal barrier in weaned piglets and IPEC-J2 cells
Source: Front Nutr. 2022 Nov 4;9:1012207. doi: 10.3389/fnut.2022.1012207 (PMC9672516; doi:10.3389/fnut.2022.1012207)
Supplement: Supplementary file 1 [file Data_Sheet_1.docx]

**Supplementary Materials**

Supplementary Table 1. Composition of diets (as-fed basis, %)^1^

|  | Dietary treatment, % | |
| --- | --- | --- |
| Item | CON | TA |
| Ingredient |  |  |
| Corn | 28.33 | 28.23 |
| Expanded corn | 30 | 30 |
| Soy protein concetrate | 6 | 6 |
| Soybean meal,43% CP | 8 | 8 |
| Fish meal,63% CP | 5 | 5 |
| Whey | 15 | 15 |
| Glucose | 3 | 3 |
| Soybean oil | 0.5 | 0.5 |
| Limestone | 0.86 | 0.86 |
| Calcium hydrogen phosphate | 0.4 | 0.4 |
| Choline chloride, 50% | 0.1 | 0.1 |
| Antioxidants^2^ | 0.05 | 0.05 |
| Citric acid | 0.8 | 0.8 |
| Salt | 0.1 | 0.1 |
| Vitamin-mineral premix^3^ | 0.45 | 0.45 |
| Tannic acid | 0 | 0.1 |
| Zinc oxide | 0.2 | 0.2 |
| L-Lysine, 98% | 0.58 | 0.58 |
| DL-Methionine | 0.38 | 0.38 |
| L-Threonine | 0.19 | 0.19 |
| L-Tryptophan | 0.06 | 0.06 |
| Total | 100 | 100 |
| Calculated composition |  |  |
| CP | 18 | 18 |
| ME, MJ/kg | 14.2 | 14.2 |
| Lysine^4^ | 1.35 | 1.35 |
| Methionine^4^ | 0.39 | 0.39 |
| Methionine + Cysteine^4^ | 0.74 | 0.74 |
| Threonine^4^ | 0.79 | 0.79 |
| Trptophane^4^ | 0.22 | 0.22 |

^1^CON, basal diet, TA, basal diet supplemented with 1000 mg/kg microencapsulated tannic acid (30% effective concentration).

^2^Antioxidants contained 60g/kg butylhydroquinone and 180g/kg ethoxyquinoline.

^3^Vitamin-mineral premix supplied per kilogram of feed: 10,000 IU of vitamin A, 1,000 IU of vitamin D_3_, 80 IU of vitamin E, 2.0 mg of vitamin K_3_, 0.03 mg of vitamin B_12_, 12 mg of riboflavin, 40 mg of niacin, 25 mg of d-pantothenic acid, 0.25 mg of biotin, 1.6 mg of folic acid, 3.0 mg of thiamine, 2.25 mg of pyridoxine, 300 mg of choline chloride, 150 mg of Fe (FeSO_4_), 100 mg of Zn (ZnSO_4_), 30 mg of Mn (MnSO_4_), 25 mg of Cu (CuSO_4_), 0.5 mg of I (KIO_3_), 0.3 mg of Co (CoSO_4_), 0.3 mg of Se (Na_2_SeO_3_), and 4.0 mg of ethoxyquin.

^4^Standardized ileal-digestible.

Supplementary Table 2. Primers used in this study

| Gene | Nucleotide sequence of primers (5’–3’) | Product length |
| --- | --- | --- |
| Keap1 | GTGTGGAGAGGAGTCTGTGTC | 112 |
|  | TCCACGTTTCTGTCTCCACG |  |
| Nrf2 | AAGTCAGAGTCGGCTGCAT | 200 |
|  | ATTGCGCAACAGATCAACAGC |  |
| Claudin-1 | CTAGTGATGAGGCAGATGAA | 250 |
|  | AGATAGGTCCGAAGCAGAT |  |
| ZO-1 | TTGATAGTGGCGTTGACA | 126 |
|  | CCTCATCTTCATCATCTTCTAC |  |
| Occludin | GAGTGATTCGGATTCTGTCT | 167 |
|  | TAGCCATAACCATAGCCATAG |  |
| TNF-α | ACAGGCCAGCTCCCTCTTAT | 102 |
|  | CCTCGCCCTCCTGAATAAAT |  |
| IL-6 | AGACCCTGAGGCAAAAGGGAAA | 209 |
|  | CGGCATCAATCTCAGGTGCC |  |
| β-actin | AGTTGAAGGTGGTCTCGTGG | 216 |
|  | TGCGGGACATCAAGGAGAAG |  |
